# Changes in body mass index, obesity, and overweight in Southern Africa development countries, 1990 to 2019: Findings from the Global Burden of Disease, Injuries, and Risk Factors Study

**DOI:** 10.1002/osp4.519

**Published:** 2021-05-13

**Authors:** Philimon N. Gona, Ruth W. Kimokoti, Clara M. Gona, Suha Ballout, Sowmya R. Rao, Chabila C. Mapoma, Justin Lo, Ali H. Mokdad

**Affiliations:** ^1^ College of Nursing & Health Sciences University of Massachusetts Boston Boston MA USA; ^2^ Department of Nutrition Simmons College Boston MA USA; ^3^ Department of Nursing MGH Institute for Health Professions Boston MA USA; ^4^ Department of Global Health Boston University School of Public Health Boston MA USA; ^5^ Department of Population Studies University of Zambia Lusaka Zambia; ^6^ Institute for Health Metrics and Evaluation University of Washington Seattle WA USA

**Keywords:** high body mass index, ischemic heart disease, mortality, overweight & obesity, prevalence, SADC countries, stroke, type 2 diabetes

## Abstract

**Background:**

High body mass index (BMI) is associated with stroke, ischemic heart disease (IHD), and type 2 diabetes mellitus (T2DM). An epidemiological analysis of the prevalence of high BMI, stroke, IHD, and T2DM was conducted for 16 Southern Africa Development Community (SADC) using Global Burden of Diseases, Injuries, and Risk Factors (GBD) Study data.

**Methods:**

GBD obtained data from vital registration, verbal autopsy, and ICD codes. Prevalence of high BMI (≥25 kg/m^2^), stroke, IHD, and T2DM attributed to high BMI were calculated. Cause of Death Ensemble Model and Spatiotemporal Gaussian regression was used to estimate mortality due to stroke, IHD, and T2DM attributable to high BMI.

**Results:**

Obesity in adult females increased 1.54‐fold from 12.0% (uncertainty interval [UI]: 11.5–12.4) to 18.5% (17.9–19.0), whereas in adult males, obesity nearly doubled from 4.5 (4.3–4.8) to 8.8 (8.5–9.2). In children, obesity more than doubled in both sexes, and overweight increased by 27.4% in girls and by 37.4% in boys. Mean BMI increased by 0.7 from 22.4 (21.6–23.1) to 23.1 (22.3–24.0) in adult males, and by 1.0 from 23.8 (22.9–24.7) to 24.8 (23.8–25.8) in adult females. South Africa 44.7 (42.5–46.8), Swaziland 33.9 (31.7–36.0) and Lesotho 31.6 (29.8–33.5) had the highest prevalence of obesity in 2019. The corresponding prevalence in males for the three countries were 19.1 (17.5–20.7), 19.3 (17.7–20.8), and 9.2 (8.4–10.1), respectively. The DRC and Madagascar had the least prevalence of adult obesity, from 5.6 (4.8–6.4) and 7.0 (6.1–7.9), respectively in females in 2019, and in males from 4.9 (4.3–5.4) in the DRC to 3.9 (3.4–4.4) in Madagascar.

**Conclusions:**

The prevalence of high BMI is high in SADC. Obesity more than doubled in adults and nearly doubled in children. The 2019 mean BMI for adult females in seven countries exceeded 25 kg/m^2^. SADC countries are unlikely to meet UN2030 SDG targets. Prevalence of high BMI should be studied locally to help reduce morbidity.

## 1 BACKGROUND

**FIGURE 1:**
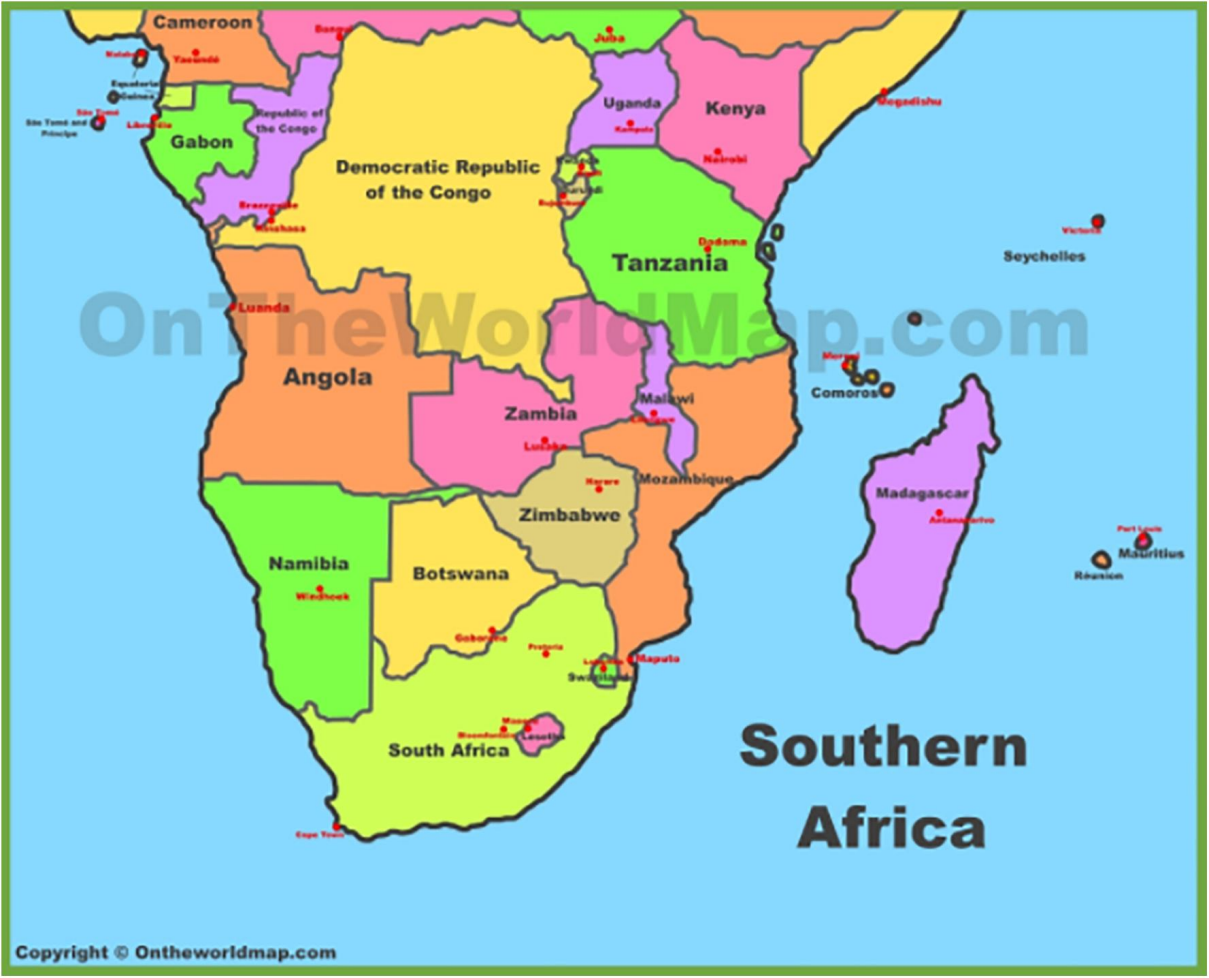
Map of Southern African Development Community Countries. Source: http://ontheworldmap.com/africa/map‐of‐southern‐africa.jpg

Approximately 39% of adults worldwide have overweight, while 13% have obesity which are known precursors for premature death and disability, resulting in low quality of life, and a wide range of health complications as well as direct and/or indirect increases in health care costs.^1^, ^2^, ^3^, ^4^, ^5^, ^6^, ^7^, ^8^, ^9^, ^10^, ^11^ Low‐ and middle‐income countries (LMICs) are experiencing a surge in the prevalence of overweight and obesity. In the past few decades, the burden of obesity has increased in LMICs, and so far, no substantial reduction has been observed in developed countries as well.^2^, ^3^, ^4^ A meta‐analysis of Demographic and Health Surveys (DHS) conducted between 2008 and 2019 across 33 countries in sub‐Saharan Africa (SSA) region of nearly 500,000 women 15 to 49 years of age revealed heterogeneity across the region in the prevalence of overweight/obesity ranging from 6.7% for Madagascar and up to 44.5% for Lesotho.^12^ The increased vulnerability to obesity and attributable conditions in the 16 SADC countries shown in Figure 1 (Angola, Botswana, Comoros, Democratic Republic of Congo [DRC], Lesotho, Madagascar, Malawi, Mauritius, Mozambique, Namibia, Seychelles, South Africa, eSwatini [formerly Swaziland], Tanzania, Zambia, and Zimbabwe) has mainly been a result of populations adapted to traditional diets now changing to rapid industrialization and urbanization, and thereby making people more susceptible to obesity, stroke, IHD, and T2DM, among other fast rising non‐communicable diseases (NCDs).^13^

The SADC countries are facing challenges similar to other developing countries, that currently have rapid demography and lifestyle changes.^14^, ^15^, ^16^, ^17^, ^18^ Five‐fold regional and seven‐fold in‐between‐country differences in the risk of stroke were observed among these countries, with the East and Central SSA, which include SADC countries, having a lifetime risk ranging from 28% to 37%.^14^ While there was a significant reduction in the risk of stroke in all Global Burden of Disease (GBD) regions and high‐income countries, and also in most countries between 1990 and 2015, there was a significant increase in the lifetime risk of stroke in seven SADC countries ranging from 4% to 32%, However, in three of the SADC countries, changes in the risk of stroke were not substantial.^14^ The prevalence of T2DM, IHD, and cardiovascular disease (CVD) increased 10‐fold in SSA between 1988 and 2008.^19^ Between 2010 and 2030, the number of adults with T2DM in developing countries is projected to increase by 69%, compared to a 20% increase in developed countries.^20^

The 16 SADC countries are a regional economic community whose aim is to increase regional socioeconomic integration to achieve greater economic growth and alleviate poverty (www.sadc.int/about‐sadc). Levels of development and poverty, social service delivery, and economic performance vary greatly among these member states. Key among the aims of SADC is the strengthening of economic cooperation and integration, providing for cross‐border investment and trade, free movement of goods and services across national borders. Although this region is at increased risk, there is no readily available systematic understanding of the distribution of obesity and overweight and time trends across all 16 SADC countries. SDG Target 3.4 aims for a one‐third reduction, relative to 2015 levels, in death rate due to CVD, cancers, chronic respiratory diseases, and diabetes by 2030 in populations aged 30–70 years.^21^ Evidence‐based information on the level and trends of BMI, obesity, and overweight is essential to support policy and goal settings, program evaluation, and decision‐making. Despite the increased burden of high BMI in the region, no comprehensive estimates of the burden exist for the SADC countries. Therefore, the goal of this analysis was to highlight the burden of obesity and overweight in SADC region and its impact on health, as well as help inform strategies and interventions to reduce high BMI in turn reducing the burden of stroke, IHD, and T2DM attributable to high BMI, thus improving the chances for attaining SDG Target 3.4. This is a descriptive, epidemiological analysis of the prevalence of high BMI, stroke, IHD, and T2DM in 1990 and 2019 for the 16 SADC countries using secondary data from the Global Burden of Diseases, Injuries and Risk Factor (GBD) Study.^22^

## 2 METHODS

The GBD is a systematic, scientific effort by the Institute for Health Metrics and Evaluation (IHME) to quantify the comparative magnitude of health loss due to diseases, injuries, and risk factors by age, sex, and geographies for specific points in time. The GBD study estimates country‐specific incidence, prevalence, mortality, years of life lost (YLLs), years lived with disability (YLDs), and disability‐adjusted life‐years (DALYs) due to diseases such as T2DM, IHD, and stroke and conditions such as high BMI. All GBD estimates adhere to the 14 Guidelines on Accurate and Transparent Health Estimate Reporting (GATHER).^23^ GATHER recommends making available statistical codes, details on the use or non‐use of specific sources, and how primary data are adjusted. In this study outcomes of interest were BMI, mortality, stroke, IHD, and T2DM morbidity attributable to high BMI.

Full details of data sources, availability of data, and methods of dietary risk factors and BMI are described in detail elsewhere,^24^, ^25^ However, to describe it briefly, GBD combines information from several sources including country‐specific surveys and administrative databases, weighting it based on quality decided by a panel of experts. Where individual‐level weight and height survey data were available the mean BMI was used to determine the prevalence of overweight and obesity. Medline for studies that provide nationally or sub‐nationally representative estimates of BMI, overweight, or obesity among children or adults were systematically searched. The search terms, selection criteria, and flow diagrams of screening are provided elsewhere,^24^ The search was conducted using the following terms: (("Body Mass Index"[Mesh] OR "Overweight"[Mesh] OR "Obesity"[Mesh]) AND "Geographic Locations"[Mesh] NOT "United States"[Mesh]) AND ("humans"[Mesh] AND "adult"[MeSH]) AND ("Data Collection"[Mesh] OR "Health Services Research"[Mesh] OR "Population Surveillance"[Mesh] OR "Vital statistics"[Mesh] OR "Population"[Mesh] OR "Epidemiology"[Mesh] OR "surve*"[TiAb]) NOT Comment[ptyp] NOT Case Reports[ptyp] NOT "hospital"[TiAb] AND ("2014/01/01"[Date ‐ Publication]:"2019/12/31"[Date ‐ Publication]) (Supplement 1).^26^ Also searched was the Global Health Data Exchange (http://ghdx.healthdata.org) for multi‐country survey programs, national surveys, and longitudinal studies that provide self‐reported or measured data on height and weight for children or adults. Full details of data sources, availability of data, and methods are described in detail elsewhere.^24^

Mean prevalence of obesity and overweight were estimated from Spatiotemporal Gaussian process regression modeling.^25^ The coefficients of this regression were applied to the prevalence of overweight and obesity to estimate the mean BMI for each country, according to age, sex, and year. Estimation was improved in data‐sparse countries using a wide range of covariates with plausible relationships to overweight and obesity. Three country‐level covariates with best fit comprising of energy intake per capita, the absolute latitude of the country, and the proportion of persons living in urban areas were included as independent variables (Supplement 1).^24^

CoD ensemble modelling (CODEm) framework was used to model cause‐specific death rates in the GBD.^27^ Mixed effects linear models and spatial‐temporal Gaussian Process Regression models were used to estimate cause fractions and death rates. The current version of the data download tool is available in the GHDx and contains core summary results for the GBD 2019: http://ghdx.healthdata.org/gbd‐results‐tool. Input data for stroke, IHD, and T2DM was obtained from a combination of vital registration, verbal autopsy, and ICD codes where available. Verbal autopsy is a validated method for determining causes of death and cause‐specific mortality fractions in populations without a complete vital registration system. Verbal autopsies consist of a trained interviewer using a questionnaire to collect information about the signs, symptoms, and demographic characteristics of a recently deceased person from an individual familiar with the deceased.^28^ The burden of disease related to high BMI for each of T2DM, IHD, and stroke attributable to high BMI was estimated by calculating the population attributable fraction (PAF) for each country, segregated by age, sex, and year.^29^ Cause of deaths due to each of the three outcomes was modeled with the CODEm approach. Covariates included in the ensemble modelling process for IHD and stroke were total cholesterol, smoking prevalence, systolic blood pressure, trans fatty acid, mean BMI, and others. Deaths attributed to Alzheimer's and other dementias, Parkinson's disease, or atrial fibrillation and flutter were not considered as stroke related. Country‐level covariates with best fit and coefficients chosen for inclusion in the ensemble modelling process for T2DM were fasting plasma glucose, prevalence of diabetes mean BMI, mean cholesterol, mean systolic blood pressure, prevalence of obesity, and others. Complete lists and description of input data, covariates used in CODEm, model specifications and processing sequence are provided online in Supplementary Appendix 1.^25^

CODEm relied on four key components: (a) all available data were identified and gathered to be used in the modelling process; (b) a diverse set of plausible models were developed to capture well‐documented associations in the estimates; (c) the out‐of‐sample predictive validity was assessed for all individual models, which were then ranked for use in the ensemble modelling stage; and (d) differently weighted combinations of individual models were evaluated to select the ensemble model with the highest out‐of‐sample predictive validity. Out‐of‐sample predictions are used to calculate mean squared error (MSE), root‐mean‐squared‐error (RMSE), coefficient of variation (CV), and 95% coverage of predictive intervals representing the proportion of observed out‐of‐sample data that fall within the predicted 95% credible intervals. The variance of the random walk was determined by estimated residuals from the out‐of‐sample validation. The out‐of‐sample 95% coverage for stroke, IHD, and T2DM all exceeded 97.5%.

The total morbidity related to high BMI was calculated as the total of disease‐specific burdens. PAF for BMI categories (20 to 24, 25 to 29, and ≥30) and each outcome, stroke, IHD, and T2DM were used to help understand locations with high concentrations of BMI burden. The Das Gupta method,^30^ details of which are described elsewhere^30^ was used to decompose the change in deaths and DALYs attributed to high BMI between population growth, population age structure, risk exposure to high BMI, and rates of risk‐deleted mortality and DALYs. Rates per 100,000 population of YLL, YLD, and DALYs attributable to high BMI were computed.

The flowchart in Figure S5 describes the process of estimating the disease burden of high BMI (Supplementary Appendix).^26^ The relative risk per 5‐unit change in BMI for each disease endpoint was obtained from meta‐analyses. Relative risks for all outcomes, by age and sex, are reported elsewhere (Table S2 of the Supplement 1).^24^

The GBD study estimates country‐specific incidence, prevalence, mortality, YLLs, YLDs, and DALYs due to stroke, IHD, and T2DM. Cause‐specific crude and age‐standardized death rates per 100,000 population were obtained from the Cause of Death Ensemble model (CODEm) and spatiotemporal Gaussian process regression (ST‐GPR). Deaths were multiplied by standard life expectancy at each 5‐year age‐group to calculate YLLs. Cause‐specific mortality was estimated using a Bayesian meta‐regression modelling tool, DisMod‐MR. Prevalence estimates were multiplied by disability weights for mutually exclusive sequelae of diseases to calculate YLDs.^24^ YLLs were calculated using the product of age‐specific life expectancy from the reference life table used in the GBD study. YLDs were calculated as a product of the prevalence of the disease‐specific disability weights.^31^, ^32^ In this calculation, risk‐deleted mortality is defined as the burden of disease in the absence of the risk factor, that is, rates of death from T2DM, IHD, and stroke assuming everyone is at the lowest‐risk BMI category. Adding together YLLs and YLDs yields DALYs. Rates per 100,000 population of YLL, YLD, and DALYs attributable to stroke, IHD, and T2DM were computed.

For changes over time, annualized rates of change (AROC) were presented as the percent difference in the natural log of the rates in 1990 and 2019 divided by 29, that is, 1 00*[ln(2019 Rate/1990 Rate)/29]. AROC (%) is a crude measure of linear trend over the 29‐year period,^22^ which when positive indicates an increasing linear trend/slope over the 29 years, a negative AROC indicates a decreasing trend/slope.

Uncertainty for each outcome was quantified using uncertainty intervals (UIs) based on 1000 bootstrap draws from the posterior distribution.^32^ UIs were determined by the 25^th^ and 975^th^ ordered values of the posterior distribution of the 1000 draws, and point estimates were computed from the mean of the draws.

The socio‐demographic index (SDI) for a country is calculated from the geometric mean of three rescaled components: total fertility rate of women under 25 years of age, lag‐distributed income per capita, and average educational attainment in the population >15 years.^33^ Global Burden of Disease Collaborative Network SDI contains an interpretable scale: 0.0 represents the lowest income per capita, lowest educational attainment, and highest total fertility rate for women under the age of 25 years, and 1.0 represents the highest income per capita, highest educational attainment, and lowest total fertility rate. Nine of the 15 SADC countries were classified as either low or low‐middle SDI (SDI range: 0.33–0.49). Mauritius (SDI: 0.72), Seychelles (SDI: 0.69), and South Africa (SDI: 0.68) were classified as high‐middle SDI.

## 3 RESULTS

Age standardized prevalence of overweight and obesity for adolescents 2–19 years and adult men and women for the years 1990 and 2019 are displayed in Table 1. Overweight in adult females increased by 8.3% from 31.4 (95% UI: 30.5–32.3) in 1990 to 39.7 (38.7–40.7 in 2019). In adult males, overweight also increased by 8.5% from 20.2 (19.5–20.8) to 28.7 (27.9–29.5) for the same period. Obesity in adult females increased by about >1.5‐fold from 12.0% (11.5–12.4) to 18.5% (17.9–19.0), while in adult males, it nearly doubled from 4.5% (4.3–4.8) to 8.8% (8.5–9.2). In children, obesity more than doubled while overweight increased by 27.4% and 37.4% in girls and boys, respectively. In 2019, South Africa [44.7% (42.5–46.8)], Swaziland [33.9% (31.7–36.0)], and Lesotho [31.6% (29.8–33.5)] had the highest prevalence of obesity in females, they also observed higher prevalence of overweight, 71.3% (68.7–73.7) for South Africa 59.6% (57.0–61.8) for Lesotho, and 62.3% (59.6–65.1) for Swaziland. In adult males, these countries had similar patterns to females, showing the highest prevalence of obesity and overweight. The DRC and Madagascar had the least prevalence of overweight and obesity. Amongst children, South Africa, and Swaziland, had the highest prevalence of obesity and overweight in the SADC (Table 1).

**TABLE 1:** Age standardized prevalence (%) of overweight and obesity among children aged 2–19 and 20 years and older in Southern Africa Development Community countries, 1990 and 2019

Country	1990				2019			
	Overweight		Obesity		Overweight		Obesity	
	Males	Females	Males	Females	Males	Females	Males	Females
2–19 years								
Angola	3.6 (3.0–4.4)	4.2 (3.4–5.3)	0.8 (0.6–1.0)	0.8 (0.6–1.1)	9.2 (7.6–11.2)	10.6 (8.6–13.2)	4.8 (3.7–6.0)	4.5 (3.4–5.8)
Botswana	4.9 (4.0–5.8)	6.7 (5.4–8.3)	1.2 (0.9–1.6)	1.8 (1.4–2.4)	9.5 (7.8–11.5)	11.6 (9.3–14.4)	5.2 (4.1–6.5)	6.8 (5.3–8.5)
Comoros	10.8 (9.0–12.9)	14.8 (12.2–18.1)	3.6 (2.8–4.5)	4.7 (3.7–5.9)	14.0 (11.7–16.4)	15.6 (12.7–18.7)	7.6 (6.1–9.4)	8.5 (6.8–10.5)
DRC	4.0 (3.3–4.8)	6.5 (5.1–8.1)	2.1 (1.6–2.6)	1.6 (1.2–2.2)	5.7 (4.7–6.9)	9.2 (7.4–11.2)	3.4 (2.7–4.2)	3.3 (2.5–4.3)
Lesotho	4.8 (3.9–5.7)	9.0 (7.2–11.2)	0.9 (0.7–1.2)	1.5 (1.1–1.9)	8.9 (7.5–10.6)	16.7 (14.0–20.0)	3.6 (2.8–4.5)	4.7 (3.7–5.8)
Madagascar	5.4 (4.5–6.5)	7.8 (6.2–9.6)	1.3 (1.0–1.6)	1.7 (1.3–2.2)	8.1 (6.7–9.7)	9.2 (7.3–11.5)	3.5 (2.7–4.4)	3.1 (2.4–4.0)
Malawi	9.9 (8.3–11.7)	11.7 (9.6–14.1)	3.0 (2.4–3.7)	3.2 (2.5–4.1)	12.9 (10.7–15.2)	14.7 (12.0–17.8)	5.8 (4.7–7.1)	6.1 (4.9–7.5)
Mauritius	6.7 (5.5–8.0)	7.9 (6.3–9.9)	2.5 (1.9–3.2)	2.8 (2.2–3.6)	13.4 (11.3–15.8)	14.5 (11.8–17.7)	10.7 (8.8–12.7)	9.3 (7.5–11.5)
Mozambique	4.6 (3.8–5.4)	7.3 (5.8–9.0)	1.0 (0.8–1.3)	1.5 (1.2–2.0)	9.8 (8.2–11.5)	14.6 (11.9–17.6)	6.2 (5.0–7.5)	7.0 (5.6–8.7)
Namibia	5.4 (4.4–6.5)	7.8 (6.2–9.5)	1.7 (1.3–2.1)	2.6 (2.0–3.4)	9.1 (7.6–10.7)	9.3 (7.6–11.3)	3.1 (2.4–3.9)	5.7 (4.5–7.0)
SADC	6.7 (6.3–7.2)	9.5 (8.8–10.3)	2.4 (2.1–2.6)	2.8 (2.5–3.1)	9.2 (8.7–9.9)	12.1 (11.3–12.9)	5.5 (5.1–5.9)	6.0 (5.5–6.4)
Seychelles	5.5 (4.5–6.7)	7.6 (6.0–9.2)	1.9 (1.5–2.4)	2.5 (1.9–3.2)	9.4 (7.7–11.2)	10.4 (8.3–12.7)	6.5 (5.2–7.9)	5.7 (4.4–7.2)
South Africa	8.8 (7.4–10.4)	13.2 (11.0–15.4)	3.6 (2.8–4.4)	5.3 (4.3–6.4)	12.1 (10.3–14.0)	17.5 (15.0–20.3)	9.2 (7.7–10.9)	13.1 (11.0–15.3)
Swaziland	11.2 (9.3–13.2)	15.6 (12.8–18.8)	3.0 (2.3–3.8)	4.7 (3.7–6.0)	14.1 (11.8–16.6)	18.5 (15.4–21.9)	7.3 (5.8–8.8)	9.7 (7.9–12.0)
Tanzania	8.5 (7.1–10.2)	10.5 (8.5–12.8)	2.3 (1.8–3.0)	2.4 (1.9–3.1)	11.3 (9.6–13.3)	11.5 (9.6–13.6)	5.4 (4.4–6.5)	5.3 (4.3–6.5)
Zambia	11.5 (9.8–13.5)	14.4 (12.0–17.4)	5.3 (4.3–6.4)	5.7 (4.5–7.1)	12.8 (10.7–15.2)	15.3 (12.5–18.3)	14.0 (11.7–16.4)	12.6 (10.4–15.0)
Zimbabwe	7.3 (6.0–8.5)	11.3 (9.3–13.7)	1.9 (1.5–2.4)	2.5 (2.0–3.2)	6.4 (5.3–7.7)	12.9 (10.5–15.7)	2.0 (1.6–2.6)	3.9 (3.1–4.9)
SADC	6.7 (6.3–7.2)	9.5 (8.8–10.3)	2.4 (2.1–2.6)	2.8 (2.5–3.1)	9.2 (8.7–9.9)	12.1 (11.3–12.9)	5.5 (5.1–5.9)	6.0 (5.5–6.4)
20+ years								
Angola	6.5 (5.8–7.3)	8.3 (7.3–9.6)	0.4 (0.3–0.4)	1.2 (1.0–1.5)	24.8 (22.8–27.0)	27.9 (24.9–30.7)	4.7 (4.0–5.3)	9.4 (8.2–10.6)
Botswana	13.1 (11.8–14.4)	25.7 (23.1–28.3)	2.2 (1.8–2.5)	10.9 (9.6–12.3)	30.3 (28.0–32.8)	43.5 (40.3–46.8)	10.8 (9.7–12.1)	27.1 (24.8–29.4)
Comoros	19.6 (17.8–21.7)	27.7 (25.0–30.5)	1.8 (1.5–2.1)	6.0 (5.1–6.9)	32.9 (30.4–35.6)	39.2 (36.0–42.6)	6.5 (5.7–7.3)	13.6 (12.1–15.2)
DRC	21.0 (19.3–22.9)	23.4 (20.9–26.0)	4.4 (3.9–5.1)	4.9 (4.3–5.7)	20.1 (18.6–21.8)	22.4 (20.0–25.0)	4.9 (4.3–5.4)	5.6 (4.8–6.4)
Lesotho	13.9 (12.5–15.3)	38.2 (35.2–41.3)	2.0 (1.7–2.3)	12.6 (11.4–14.1)	28.8 (27.0–30.7)	59.6 (57.0–61.8)	9.2 (8.4–10.1)	31.6 (29.8–33.5)
Madagascar	9.8 (8.9–10.7)	16.0 (14.5–17.6)	1.3 (1.1–1.5)	3.5 (3.0–3.9)	20.0 (18.1–21.9)	23.2 (20.8–25.4)	3.9 (3.4–4.4)	7.0 (6.1–7.9)
Malawi	14.5 (13.0–16.0)	18.7 (16.7–20.9)	1.6 (1.3–1.8)	3.6 (3.1–4.2)	27.7 (25.5–30.0)	35.2 (32.0–38.4)	5.6 (4.9–6.3)	11.3 (10.1–12.6)
Mauritius	25.8 (23.7–28.0)	31.8 (29.1–34.7)	4.0 (3.5–4.6)	10.9 (9.7–12.1)	49.8 (47.2–52.5)	54.1 (51.1–57.3)	14.6 (13.4–15.8)	22.2 (20.6–23.9)
Mozambique	7.2 (6.4–8.0)	13.2 (11.6–14.9)	0.7 (0.6–0.8)	2.4 (2.0–2.8)	23.6 (21.6–25.7)	35.2 (32.1–38.3)	5.5 (4.7–6.2)	12.0 (10.6–13.6)
Namibia	21.3 (19.5–23.2)	35.2 (32.1–38.2)	4.7 (4.1–5.4)	13.9 (12.4–15.5)	29.4 (27.4–31.4)	45.0 (42.4–47.4)	10.2 (9.1–11.3)	23.0 (21.4–24.7)
Seychelles	27.4 (25.8–29.0)	45.1 (43.0–47.2)	4.7 (4.3–5.2)	19.1 (18.0–20.2)	46.3 (43.8–48.7)	53.7 (50.8–56.5)	15.5 (14.2–16.9)	25.7 (23.8–27.5)
South Africa	32.2 (30.6–34.0)	57.0 (55.0–59.2)	9.6 (8.8–10.5)	29.5 (27.8–31.1)	44.9 (42.4–47.3)	71.3 (68.7–73.7)	19.3 (17.7–20.8)	44.7 (42.5–46.8)
Swaziland	36.9 (34.3–39.7)	55.5 (52.1–58.9)	9.4 (8.4–10.6)	24.2 (22.2–26.2)	51.6 (48.9–54.1)	62.3 (59.6–65.1)	19.1 (17.5–20.7)	33.9 (31.7–36.0)
Tanzania	17.7 (16.1–19.3)	25.7 (23.1–28.3)	2.9 (2.5–3.3)	7.4 (6.5–8.4)	28.8 (26.8–31.0)	37.3 (34.7–40.1)	8.3 (7.5–9.2)	16.7 (15.3–18.1)
Zambia	20.0 (18.2–22.1)	23.9 (21.3–26.4)	3.7 (3.1–4.3)	6.8 (5.9–7.8)	31.9 (29.1–34.5)	35.0 (32.1–37.9)	8.8 (7.7–10.0)	13.8 (12.3–15.2)
Zimbabwe	18.9 (17.2–20.7)	38.9 (35.8–42.1)	3.5 (3.1–4.0)	14.7 (13.2–16.2)	20.1 (18.7–21.5)	45.1 (42.8–47.2)	4.8 (4.3–5.3)	19.3 (18.0–20.7)
SADC	20.2 (19.5–20.8)	31.4 (30.5–32.3)	4.5 (4.3–4.8)	12.0 (11.5–12.4)	28.7 (27.9–29.5)	39.7 (38.7–40.7)	8.8 (8.5–9.2)	18.5 (17.9–19.0)

### 3.1 Mean BMI

Between 1990 and 2019, age‐standardized mean SADC countries increased from 22.4 (21.6–23.1) to 23.1 (22.3–24.0) in adult males, an increase of 0.7, while it increased by 1.0 from 23.8 (22.9–24.7) to 24.8 (23.8–25.8) in adult females suggesting that a typical adult in SADC has normal weight generally. However, in 2019 a typical adult male in Swaziland was overweight, 26.2 (25.0–27.4), and a typical adult woman in 5 of the 16 countries (South Africa, Swaziland, Botswana, Lesotho, Seychelles) had overweight with mean BMI ranging from 26.4 (25.2–27.8) in Seychelles, to 29.5 (27.7–31.5) in South Africa. Madagascar had the lowest mean BMI in 2019 with 21.4 (20.8–22.2) in adult males and 21.9 (21.2–22.7) in adult females. BMI increase exceeded two units in both adult men and women in Botswana, and Mauritius, in South Africa and Botswana, BMI increases exceeded two units in adult women but not in adult men. There was a positive linear association between the change in BMI from 1990 to 2019 in males and females, both with Pearson correlation coefficient *r* = 0.74, *p* < 0.001 suggesting that countries with the greatest increase in mean BMI in females tended to be the same countries with the greatest increases in mean BMI in males as well. Notably, the DRC and Madagascar did not experience an increase in BMI in both adult females and adult males (Table 2).

**TABLE 2:** Age standardized mean BMI among adult males and females in Southern Africa Development Community countries, 1990 and 2019

Country	Adult males			Adult females		
	1990	2019	BMI change^a^	1990	2019	BMI change^a^
Angola	21.3 (20.7–22.0)	22.7 (21.9–23.5)	+1.4	21.6 (21.0–22.3)	23.2 (22.4–24.0)	+1.6
Botswana	21.8 (21.2–22.5)	24.1 (23.2–25.0)	+2.3	23.9 (23.0–24.9)	27.5 (26.2–28.8)	+3.6
Comoros	22.4 (21.7–23.1)	23.4 (22.5–24.2)	+1.0	23.2 (22.3–24.0)	24.2 (23.3–25.3)	+1.0
DRC	22.0 (21.3–22.8)	22.0 (21.3–22.7)	0.0	22.3 (21.6–23.1)	22.3 (21.5–23.1)	0.0
Lesotho	21.9 (21.3–22.6)	23.4 (22.6–24.2)	+1.5	24.4 (23.5–25.4)	27.5 (26.1–29.0)	+3.1
Madagascar	20.7 (20.1–21.4)	21.4 (20.8–22.2)	+0.7	21.3 (20.7–22.0)	21.9 (21.2–22.7)	+0.6
Malawi	22.2 (21.5–22.9)	23.3 (22.5–24.1)	+1.1	22.7 (22.0–23.5)	24.3 (23.4–25.3)	+1.6
Mauritius	22.5 (21.7–23.3)	24.7 (23.7–25.8)	+2.2	23.4 (22.6–24.3)	25.7 (24.4–26.9)	+2.3
Mozambique	21.4 (20.7–22.0)	22.5 (21.8–23.3)	+1.1	21.9 (21.2–22.6)	23.6 (22.7–24.6)	+1.7
Namibia	21.8 (21.1–22.5)	22.9 (22.1–23.8)	+1.1	23.8 (22.9–24.8)	25.5 (24.4–26.6)	+1.7
Seychelles	23.3 (22.5–24.1)	25.1 (24.1–26.2)	+1.8	25.4 (24.4–26.5)	26.4 (25.2–27.8)	+1.0
South Africa	23.9 (23.0–24.8)	25.4 (24.3–26.6)	+1.5	27.2 (25.9–28.6)	29.5 (27.7–31.5)	+2.3
Swaziland	24.5 (23.5–25.5)	26.2 (25.0–27.4)	+1.7	27.0 (25.7–28.3)	28.3 (26.9–29.9)	+1.3
Tanzania	22.0 (21.4–22.8)	23.2 (22.3–24.0)	+1.2	23.0 (22.2–23.8)	24.4 (23.4–25.5)	+1.4
Zambia	22.5 (21.8–23.3)	23.6 (22.7–24.5)	+1.1	23.1 (22.3–23.9)	24.3 (23.4–25.3)	+1.2
Zimbabwe	21.9 (21.2–22.7)	22.1 (21.4–22.9)	+0.2	24.5 (23.6–25.6)	25.5 (24.4–26.6)	+1.0
SADC	22.4 (21.6–23.1)	23.1 (22.3–24.0)	+0.7	23.8 (22.9–24.7)	24.8 (23.8–25.8)	+1.0

^a^ Pearson correlation between change in adult males and females = +0.74, *p* = 0.001.

### 3.2 Rates of deaths, YLLs, YLDs, and DALYs due to high BMI

Age‐standardized mortality attributed to high BMI increased by 1.15% annually for adult males from 57.3 (28.8–94.6) in 1990 to 80.0 (47.2–118.7) in 2019 in SADC countries. Among adult males in 2019, six countries (Mauritius, Lesotho, Botswana, Namibia, South Africa, and Swaziland) had mortality rates exceeding 100 per 100,000. Among females, in 2019, seven countries (Botswana, Lesotho, Mauritius, Namibia, South Africa, Swaziland, and Zimbabwe) had mortality rates exceeding 100 per 100,000). In adult females the AROC (%) was 0.64 from 69.9 (42.9–104.7) in 1990 to 84.1 (54.9–119.3) in 2019. Mozambique had a 3.28‐fold increase (AROC = 3.1%) followed by Lesotho with a 3.26‐fold increase (AROC = 4.08%) in males, while in adult females Lesotho had 3.08‐fold increase (AROC = 2.44%) followed by Mozambique with 3.01‐fold increase (AROC = 2.40%). Only DRC experienced a decline in mortality rate per 100,000 attributable to high BMI. in adult males from 80.5 (40.6–125.6) in 1990 to 67.2 (34.2–106.1) in 2019, while among adult females it reduced from 64.7 (31.1–105.9) in 1990 to 54.3 (25.6–93.4) in 2019. These declines correspond to an AROC of −0.62 and −0.60 in males and females, respectively. Mortality attributed to high BMI was higher in males than females in half the SADC countries. Similar patterns were observed for YLLs and DALYs attributed to high BMI (Table 3). Table 4 displays absolute number of deaths and DALYs for IHD, stroke, and T2DM attributable to high BMI, for 1990 and 2019 in all 16 SADC countries. Overall, 24,520, 34,260, and 33,940 deaths due to IHD, stroke, and T2DM, respectively, attributable to high BMI occurred in 2019 as compared to 8,850, 13,850, and 96,90 deaths due to IHD, stroke, and T2DM, respectively, in 1990, or corresponding to a 2.5‐3.5‐fold increase. Overall, DALYs were 689,460 for IHD, 1,167,470 for stroke, and 1,350,700 for T2DM attributable to high BMI in 2019 (Table 4).

**TABLE 3:** Age‐standadized mortality, YLLs, and DALYs per 100,000 population due to high BMI in Southern Africa Development Community countries, 1990 and 2019

Country	Rate per 100,000 population			Rate per 100,000 population		
Death rate	1990 Males	2019 Males	AROC	1990 Females	2019 Females	AROC
Angola	30.5 (6.0–76.3)	65.1 (30.1–105.6)	2.61	27.7 (6.1–66.4)	60.7 (31.2–97.9)	2.71
Botswana	50.1 (15.5–100.2)	140.3 (79.8–210.8)	3.55	101.1 (55.5–164.6)	183.0 (122.2–256.6)	2.05
Comoros	42.8 (12.0–86.0)	53.4 (25.4–87.0)	0.76	65.1 (26.6–114.5)	73.9 (41.7–114.9)	0.44
DRC	80.5 (40.6–125.6)	67.2 (34.2–106.1)	−0.62	64.7 (31.1–105.9)	54.3 (25.6–93.4)	0.60
Lesotho	50.0 (16.4–94.8)	163.2 (94.9–241.4)	4.08	85.4 (51.5–128.2)	208.4 (124.2–305.6)	3.08
Madagascar	33.6 (10.3–67.0)	57.7 (26.7–98.0)	1.86	57.4 (26.9–97.8)	90.3 (45.8–143.4)	1.56
Malawi	31.9 (8.2–71.6)	73.1 (34.5–116.4)	2.86	35.3 (10.0–75.7)	59.2 (31.3–93.9)	1.78
Mauritius	103.5 (52.1–163.6)	137.9 (84.1–200.7)	0.99	98.9 (59.8–139.8)	118.2 (76.6–165.8)	0.61
Mozambique	23.4 (4.6–57.3)	76.8 (34.2–130.1)	4.10	25.8 (6.6–57.3)	61.8 (30.7–102.6)	3.01
Namibia	73.8 (37.2–119.0)	114.9 (68.2–167.6)	1.53	110.7 (69.6–160.9)	117.8 (77.8–166.8)	0.21
Seychelles	73.9 (31.0–127.4)	83.9 (49.4–125.3)	0.44	69.8 (41.2–104.8)	71.6 (43.3–105.6)	0.09
South Africa	80.7 (49.8–116.4)	126.5 (89.5–167.7)	1.55	114.9 (84.2–147.2)	136.1 (100.9–170.7)	0.58
Swaziland	152.3 (90.6–223.4)	283.8 (191.0–390.0)	2.15	178.1 (124.2–238.0)	208.6 (128.1–306.0)	0.55
Tanzania	44.4 (16.4–81.2)	66.4 (35.7–101.0)	1.39	59.2 (29.1–98.7)	82.7 (52.3–119.0)	1.15
Zambia	46.2 (16.3–87.0)	91.3 (47.2–143.6)	2.35	59.2 (24.9–105.2)	90.2 (52.1–137.2)	1.45
Zimbabwe	37.9 (14.7–67.9)	59.2 (28.4–97.5)	1.54	81.8 (50.4–117.6)	121.4 (76.1–178.3)	1.36
SADC	57.3 (28.8–94.6)	80.0 (47.2–118.7)	1.15	69.9 (42.9–104.7)	84.1 (54.9–119.3)	0.64
YLLs						
Angola	818.4 (166.4–2017.0)	1673.8 (805.8–2690.1)	2.47	696.6 (156.3–1600.6)	1472.3 (774.2–2267.3)	0.58
Botswana	1274.6 (393.3–2552.9)	3554.0 (2033.2–5305.7)	3.54	2475.3 (1396.6–4001.0)	4001.1 (2635.2–5663.3)	1.66
Comoros	1133.2 (315.0–2225.5)	1347.3 (667.2–2127.5)	0.60	1604.5 (626.6–2808.5)	1765.2 (1056.4–2650.8)	−0.33
DRC	2010.5 (1039.2–3183.9)	1632.8 (818.1–2588.1)	−0.72	1605.8 (821.0–2587.5)	1330.7 (666.1–2195.1)	−0.65
Lesotho	1234.8 (409.6–2319.2)	4219.4 (2455.0–6298.5)	–	2011.3 (1258.6–2949.7)	4772.6 (2811.6–6966.8)	–
Madagascar	968.8 (308.7–1900.0)	1606.5 (766.7–2676.5)	1.74	1527.4 (738.9–2518.4)	2289.7 (1185.5–3553.4)	1.40
Malawi	846.5 (228.8–1836.2)	1765.3 (826.1–2803.0)	2.53	908.4 (284.7–1830.4)	1351.5 (755.1–2058.8)	1.37
Mauritius	2861.4 (1498.8–4375.9)	3502.2 (2200.6–5018.3)	0.70	2451.1 (1543.5–3372.1)	2668.8 (1775.5–3728.7)	0.29
Mozambique	605.8 (127.3–1434.7)	2120.1 (987.5–3493.0)	4.32	637.8 (183.0–1350.7)	1500.7 (780.3–2392.5)	2.95
Namibia	1880.6 (937.9–3041.4)	2830.4 (1727.6–4118.6)	1.41	2584.9 (1664.5–3611.1)	2407.7 (1598.3–3427.6)	−0.24
Seychelles	2096.2 (945.4–3371.5)	2295.1 (1447.1–3274.6)	–	1862.4 (1221.6–2586.6)	1706.3 (1122.6–2369.6)	–
South Africa	2209.0 (1423.1–3103.2)	2936.2 (2150.5–3807.4)	0.98	2784.2 (2117.3–3437.9)	2737.8 (2094.8–3328.1)	−0.06
Swaziland	3759.5 (2288.1–5475.3)	6997.3 (4679.2–9678.1)	2.14	3924.8 (2723.2–5239.4)	4322.2 (2574.0–6488.2)	0.33
Tanzania	1140.1 (427.3–2006.3)	1577.7 (865.9–2421.0)	1.12	1404.3 (735.4–2229.7)	1925.0 (1277.2–2630.4)	1.09
Zambia	1245.1 (463.0–2249.7)	2375.5 (1274.6–3680.0)	2.23	1553.7 (702.8–2647.0)	2187.4 (1332.4–3203.9)	1.18
Zimbabwe	985.0 (398.7–1703.4)	1551.0 (745.6–2497.3)	1.57	1911.2 (1239.1–2648.1)	2899.4 (1826.1–4186.1)	1.44
SADC	1503.2 (782.0–2384.5)	1976.7 (1188.5–2867.0)	–	1710.5 (1102.3–2464.3)	1929.8 (1297.1–2665.0)	–
DALYs						
Angola	916.5 (188.4–2208.2)	2002.4 (965.7–3157.6)	2.70	800.8 (187.7–1820.1)	1806.5 (1000.9–2728.5)	2.81
Botswana	1416.0 (440.2–2769.2)	4042.8 (2346.1–6042.0)	3.62	2840.0 (1636.4–4478.6)	4800.0 (3348.1–6504.1)	1.81
Comoros	1290.5 (375.5–2505.5)	1627.2 (835.6–2526.0)	0.80	1833.7 (761.0–3135.7)	2104.4 (1280.9–3128.7)	0.47
DRC	2256.0 (1187.3–3510.5)	1944.2 (1005.8–3023.8)	−0.51	1824.0 (950.0–2883.6)	1592.3 (827.2–2550.3)	−0.47
Lesotho	1367.3 (455.7–2553.1)	4610.8 (2716.6–6872.2)	–	2338.3 (1465.4–3344.3)	5443.6 (3365.5–7757.6)	–
Madagascar	1072.2 (344.2–2076.5)	1830.4 (888.6–3014.2)	1.84	1705.6 (828.4–2824.6)	2600.0 (1383.4–3938.0)	1.45
Malawi	965.0 (266.3–2051.3)	2073.0 (981.7–3249.4)	2.64	1060.1 (338.1–2103.9)	1702.4 (990.7–2553.1)	1.63
Mauritius	3230.0 (1686.0–4911.3)	4339.7 (2776.0–6090.5)	1.02	2995.8 (1893.5–4100.0)	3666.6 (2527.1–4954.3)	0.70
Mozambique	684.1 (144.8–1648.8)	2397.5 (1121.2–3912.9)	4.32	744.8 (220.0–1543.8)	1833.2 (1000.8–2820.5)	3.11
Namibia	2101.3 (1061.8–3339.5)	3233.2 (1980.8–4640.9)	1.49	3003.8 (2004.4–4143.2)	2996.5 (2091.6–4105.6)	−0.01
Seychelles	2393.1 (1087.0–3864.2)	3019.6 (1949.8–4266.9)	–	2370.6 (1578.1–3293.1)	2606.4 (1765.7–3544.9)	–
South Africa	2562.7 (1663.1–3550.3)	3545.3 (2609.9–4590.3)	1.12	3417.7 (2590.3–4237.9)	3594.0 (2794.8–4376.4)	0.17
Swaziland	4148.5 (2557.7–5953.1)	7702.3 (5191.2–10,484.5)	2.13	4531.7 (3214.9–5993.7)	5156.7 (3252.4–7415.9)	0.45
Tanzania	1274.5 (480.2–2241.8)	1874.8 (1049.2–2834.4)	1.33	1609.1 (853.4–2521.2)	2337.7 (1590.6–3178.8)	1.29
Zambia	1392.0 (522.4–2499.9)	2693.0 (1468.2–4107.3)	2.28	1757.6 (806.2–2972.7)	2570.3 (1611.7–3685.5)	1.31
Zimbabwe	1152.9 (472.1–1972.7)	1814.2 (888.5–2898.0)	1.56	2307.8 (1540.4–3165.3)	3555.7 (2337.8–4952.7)	1.49
SADC	1705.9 (894.5–2693.0)	2328.7 (1429.7–3332.3)	–	2013.3 (1308.3–2886.9)	2369.4 (1633.8–3250.0)	–

**TABLE 4:** Deaths and DALYs for IHD, stroke, and T2DM attributable to high BMI, 1990 and 2019 in SADC countries

SADC	Cause	Age group	1990 Males	2019 Males	1990 Females	2019 Females
Deaths	IHD	15 ≤ 49	980 (460–1710)	2550 (1230–4390)	700 (390–1120)	1360 (710–2280)
		50 ≤ 69	2410 (1100–4170)	6770 (3480–10,920)	2060 (1100–3330)	5600 (3180–8770)
		≥70	1000 (370–1960)	3050 (1430–5250)	1700 (790–2980)	5190 (2600–8620)
	Stroke	15 ≤ 49	1640 (760–2770)	4090 (2030–6800)	2020 (1140–3140)	3540 (1970–5610)
		50 ≤ 69	3320 (1430–5920)	8900 (4560–14,390)	4100 (2200–6550)	9870 (5730–14,910)
		≥70	990 (350–2000)	2700 (1210–4730)	1780 (780–3180)	5160 (2500–8660)
	T2DM	15 ≤ 49	610 (330–960)	1960 (1140–2970)	690 (450–990)	1470 (920–2150)
		50 ≤ 69	2460 (1290–3960)	9070 (5650–13,040)	3110 (2020–4370)	9840 (6900–13,100)
		≥70	960 (380–1810)	3980 (2150–6260)	1860 (1040–2920)	7620 (4660–11,010)
DALYs	IHD	15 ≤ 49	47,690 (22,350–83,040)	125,300 (60,450–216,280)	35,150 (19,530–55,920)	67,260 (35,190–111,820)
		50 ≤ 69	73,840 (34,140–127,090)	207,070 (107,430–332,820)	61,580 (33,320–98,520)	166,270 (95,240–259,380)
		≥70	16,550 (6240–32,310)	49,160 (23,330–84,450)	25,270 (12,200–44,200)	74,400 (38,390–121,630)
	Stroke	15 ≤ 49	86,760 (40,280–146,320)	219,160 (111,490–357,590)	113,270 (65,130–172,000)	204,980 (120,940–310,220)
		50 ≤ 69	105,060 (45,960–184,770)	282,040 (146,690–453,870)	133,830 (73,050–210,800)	322,780 (190,760–480,100)
		≥70	17,870 (6350–35,820)	48,200 (21,890–84,000)	31,820 (14,340–55,440)	90,310 (45,620–147,200)
	T2DM	15 ≤ 49	42,370 (22,800–65,550)	159,820 (94,300–233,470)	54,560 (35,430–77,250)	154,220 (103,810–214,790)
		50 ≤ 69	94,350 (50,560–148,050)	372,460 (236,070–525,200)	122,630 (82,330–168,170)	425,670 (306,280–558,830)
		≥70	20,640 (8300–38,440)	85,560 (46,950–133,140)	38,280 (21,970–58,500)	152,970 (98,010–215,810)

Table 5 shows the results of mortality rates due to IHD, stroke, and T2DM attributed to high BMI among persons 15 years or older. Mortality due to IHD increased annually by at least 2% in both men and women in Mozambique and Angola, however, in Botswana, this change was only observed in females. Mortality due to IHD decelerated in males and in females in the DRC and Mauritius. Mortality due to stroke attributed to high BMI increased annually by at least 2% in both men and women in two countries namely, Mozambique and Angola. In Botswana, the annual increase exceeded 2%. Mortality for stroke decelerated in both men and women in the DRC. In eSwatini, there was an observed deceleration in mortality rate for stroke in women but not in men. The mortality rate due to T2DM attributed to high BMI increased by at least 2% annually in both men and women in five countries (Mozambique, Botswana, Zimbabwe, Angola, and Mauritius). There was a greater than 2% annual increase in men in eSwatini and Zambia but not in women. Only in DRC there was deceleration in both men and women.

**TABLE 5:** Death rates 100,000 population attributable to high BMI among those who died from IHD, stroke, and T2DM, by age groups for persons 15 years or older in SADC countries, 1990 and 2019

Country	Cause	Age	Males death rate/100,000 population			Females death rate per 100,000 population		
			1990	2019	AROC%	1990	2019	AROC%
Angola	IHD	15 ≤ 49	1.3 (0.2–3.3)	2.7 (1.2–4.9)	2.69	0.5 (0.1–1.4)	1.2 (0.5–4.9)	2.99
		50 ≤ 69	19.2 (3.6–49.0)	39.6 (17.3–69.2)	2.50	11.8 (2.5–29.1)	29.8 (13.9–51.2)	3.20
		≥70	31.3 (4.8–90.1)	75.1 (27.2–142.3)	3.02	35.1 (6.0–96.0)	86.4 (32.2–166.5)	3.11
	Stroke	15 ≤ 49	2.3 (0.4–5.6)	4.3 (1.9–7.3)	2.13	2.0 (0.4–4.9)	3.4 (1.6–5.7)	1.90
		50 ≤ 69	32.3 (6.2–77.6)	57.3 (26.0–96.4)	1.97	25.1 (5.3–60.6)	47.9 (23.8–78.4)	2.23
		≥70	40.3 (6.1–113.1)	79.2 (28.2–148.9)	2.33	38.8 (6.1–104.4)	77.5 (29.8–147.8)	2.39
	T2DM	15 ≤ 49	0.9 (0.2–2.2)	2.1 (1.0–3.4)	3.04	0.6 (0.1–1.3)	1.4 (0.7–2.2)	3.06
		50 ≤ 69	20.3 (4.1–47.9)	48.6 (23.0–77.6)	3.01	11.5 (2.6—26.2)	31.2 (17.1–48.5)	3.45
		≥70	30.9 (4.9–85.0)	87.9 (31.8–164.0)	3.61	18.9 (3.5–49.6)	54.8 (22.3–101.1)	3.61
Comoros	IHD	15 ≤ 49	1.8 (0.3–3.8)	3.2 (1.3–5.7)	2.0	1.2 (0.3–2.5)	2.7 (1.3–4.7)	2.67
		50 ≤ 69	25.7 (6.9–53.3)	33.9 (14.4–61.0)	0.95	19.1 (6.1–37.7)	31.6 (15.7–54.0)	1.73
		≥70	52.2 (12.3–121.6)	75.5 (25.4–148.6)	1.27	73.8 (25.8–145.3)	105.0 (43.6–196.2)	1.22
	Stroke	15 ≤ 49	3.9 (0.8–7.9)	4.3 (2.0–7.8)	0.39	5.7 (1.5–10.4)	5.9 (3.1–9.4)	0.10
		50 ≤ 69	43.0 (11.7–90.6)	40.1 (17.4–70.3)	−0.24	58.4 (19.0–111.2)	55.3 (29.4–86.6)	−0.19
		≥70	56.8 (12.8–131.0)	62.6 (21.8–124.1)	0.34	110.5 (37.9–219.5)	106.4 (45.8–188.8)	−0.13
	T2DM	15 ≤ 49	1.0 (0.2–2.0)	1.6 (0.8–2.8)	1.66	1.5 (0.4–2.8)	2.0 (1.2–3.1)	1.16
		50 ≤ 69	22.4 (6.3–44.8)	30.2 (14.7–49.9)	1.04	12.8 (9.6–45.1)	33.7 (20.2–50.7)	0.92
		≥70	40.7 (10.0–90.1)	66.4 (24.1–120.5)	1.69	63.7 (24.4–116.2)	82.2 (38.4–137.9)	1.69
Botswana	IHD	15 ≤ 49	1.6 (0.4–3.8)	6.3 (2.7–12.0)	4.68	1.4 (0.5–2.6)	4.2 (2.1–7.2)	3.95
		50 ≤ 69	32.5 (9.1–68.1)	97.6 (49.0–162.3)	3.78	43.0 (20.1–73.8)	69.5 (38.0–110.8)	1.66
		≥70	60.0 (17.2–133.2)	161.8 (72.3–282.7)	3.42	102.2 (38.5–195.5)	255.5 (141.8–411.9)	3.16
	Stroke	15 ≤ 49	2.8 (0.8–6.1)	6.6 (3.1–12.0)	2.93	4.2 (1.8–7.2)	6.1 (3.2–10.0)	1.31
		50 ≤ 69	47.4 (13.5–98.2)	97.5 (49.9–159.3)	2.48	102.2 (50.9–67.6)	98.6 (57.0–151.8)	−0.12
		≥70	69.0 (19.2–151.0)	140.9 (59.4–254.2)	2.46	148.8 (58.3–281.3)	250.0 (143.5–397.6)	1.79
	T2DM	15 ≤ 49	0.9 (0.3–1.9)	4.6 (2.5–7.4)	5.51	1.6 (0.8–2.7)	4.3 (2.3–6.9)	3.14
		50 ≤ 69	34.7 (10.5–70.6)	137.4 (77.9–207.4)	4.75	90.4 (48.2–146.0)	157.9 (96.3–237.5)	1.92
		≥70	65.0 (18.1–139.1)	256.5 (123.9–439.7)	4.73	155.6 (66.5–285.9)	466.8 (294.3–700.7)	4.73
DRC	IHD	15 ≤ 49	2.4 (1.0–4.4))	2.1 (0.8–4.1)	−0.38	1.2 (0.5–2.5)	1.1 (0.4–2.2)	−0.41
		50 ≤ 69	48.1 (21.6–83.4)	36.2 (16.2–62.3)	−0.98	31.3 (13.2–58.1)	27.9 (12.2–53.5)	−0.40
		≥70	96.0 (38.3–178.6)	81.9 (34.2–146.3)	−0.55	87.4 (31.2–179.8)	72.0 (23.8–152.4)	−0.67
	Stroke	15 ≤ 49	3.9 (1.8–6.8)	3.3 (1.3–5.9)	−0.58	4.1 (1.8–7.4)	2.9 (1.3–5.1)	−1.15
		50 ≤ 69	71.5 (33.4–121.1)	52.8 (24.6–89.4)	−1.04	91.5 (26.8–101.2)	45.3 (20.4–80.5)	−0.85
		≥70	109.5 (43.3–208.4)	86.4 (34.9–162.162.2)	−0.82	91.5 (32.6–181.6)	64.2 (20.7–132.2)	−1.22
	T2DM	15 ≤ 49	2.0 (1.0–3.2)	1.8 (0.8–3.0)	−0.32	1.3 (0.6–2.1)	1.0 (0.5–1.7)	−0.74
		50 ≤ 69	55.2 (29.6–87.7)	43.9 (22.4–69.7)	−0.79	24.1 (12.7–40.1)	21.0 (11.0–33.5)	−0.48
		≥70	103.9 (42.4–194.6)	94.1 (40.7–167.9)	−0.34	41.5 (16.1–77.7)	33.4 (11.5–68.3)	−0.34
Lesotho	IHD	15 ≤ 49	1.0 (0.3–2.2)	5.1 (2.3–9.2)	–	0.8 (0.4–1.5)	3.3 (1.6–5.8)	–
		50 ≤ 69	25.3 (7.7–51.6)	80.2 (41.0–133.7)	–	25.4 (12.8–43.3)	70.0 (35.4–112.4)	–
		≥70	48.8 (13.3–109.1)	123.6 (56.5–211.6)	–	79.8 (35.0–144.7)	205.0 (107.3–336.2)	–
	Stroke	15 ≤ 49	2.2 (0.6–4.4)	8.1 (3.8–13.8)	–	3.3 (1.8–5.2)	7.0 (3.8–11.3)	–
		50 ≤ 69	49.0 (15.3–96.7)	127.4 (66.4–206.8)	–	77.5 (41.9–121.5)	138.8 (74.9–216.0)	–
		≥70	74.0 (20.2–157.7)	169.0 (74.295.4)	–	136.9 (69.1–248.2)	292.2 (150.9–482.1)	–
	T2DM	15 ≤ 49	0.8 (0.2–1.6)	5.2 (2.8–8.7)	–	1.2 (0.7–1.9)	4.6 (2.4–7.3)	–
		50 ≤ 69	35.6 (12.4–69.4)	161.0 (90.4–244.3)	–	68.2 (42.4–101.3)	204.5 (116.5–305.2)	–
		≥70	68.7 (20.0–141.7)	274.5 (131.5–459.5)	–	131.8 (64.4–220.2)	469.7 (260.260.8–714.6)	–
Madagascar	IHD	15 ≤ 49	2.0 (0.6–4.2)	3.7 (1.5–6.9)	2.03	0.9 (0.4–1.7)	1.4 (0.5–2.7)	1.47
		50 ≤ 69	18.8 (5.2–5.2–38.8)	37.7 (14.2–71.3)	2.40	13.6 (5.6–25.0)	31.3 (14.0–52.7)	()2.88
		≥70	41.8 (9.6–96.7)	72.9 (25.7–145.8)	1.92	46.3 (16.8–90.6)	82.6 (30.5–155.7)	1.99
	Stroke	15 ≤ 49	5.9 (1.7–11.5)	8.1 (3.5–14.7)	1.13	7.3 (3.1–12.9)	8.0 (3.6–14.1)	0.32
		50 ≤ 69	38.6 (11.2–79.5)	65.7 (26.7–122.0)	1.83	59.6 (26.3—105.3)	106.9 (54.6–168.1)	2.02
		≥70	59.6 (26.3–105.3)	74.5 (26.5–147.9)	1.81	80.7 (30.2–160.5)	141.4 (51.0–264.5)	1.93
	T2DM	15 ≤ 49	0.7 (0.2–1.3)	1.1 (0.5–1.9)	1.67	1.1 (0.5–2.0)	1.2 (0.6–2.1)	0.28
		50 ≤ 69	12.1 (3.6–25.1)	23.2 (10.4–41.4)	2.25	18.8 (8.7–31.1)	31.9 (17.2–49.2)	1.83
		≥70	21.7 (5.1–52.9)	40.7 (15.2–83.2)	2.16	35.3 (14.2–68.3)	59.7 (23.9–109.0)	2.16
Malawi	IHD	15 ≤ 49	1.6 (0.4–3.4)	2.7 (1.1–4.9)	1.89	1.1 (0.4–2.2)	1.2 (0.5–2.0)	0.04
		50 ≤ 69	17.9 (4.2–43.1)	37.8 (15.0–67.7)	2.57	13.3 (3.2–29.7)	23.8 (11.6–40.2)	2.01
		≥70	34.6 (6.9–87.9)	97.5 (40.5–179.6)	3.57	35.6 (7.3–89.1)	71.3 (27.4–132.4)	0.94
	Stroke	15 ≤ 49	2.9 (0.9–5.6)	4.3 (1.8–7.4)	1.42	3.1 (1.1–5.8)	2.8 (1.7–4.5)	−0.46
		50 ≤ 69	29.2 (6.9–67.7)	54.3 (21.7–97.4)	2.14	29.3 (7.4–62.4)	45.1 (23.3–72.8)	1.49
		≥70	44.5 (8.8–111.0)	109.5 (46.2–193.6)	3.11	51.0 (9.9–121.9)	82.2 (30.8–154.1)	1.64
	T2DM	15 ≤ 49	1.0 (0.3–1.9)	1.7 (0.7–2.8)	1.90	1.1 (0.4–1.9)	0.9 (0.5–1.5)	−0.44
		50 ≤ 69	18.4 (4.6–40.4)	41.3 (18.2–66.4)	2.78	16.7 (4.8–34.0)	27.5 (15.9–41.0)	1.72
		≥70	36.8 (7.4–87.5)	112.7 (50.0–183.7)	3.86	35.0 (7.3–83.3)	65.2 (26.7–115.7)	3.86
Mauritius	IHD	15 ≤ 49	11.5 (5.5–18.3)	9.8 (5.7–14.6)	−0.55	4.1 (2.2–6.0)	2.7 (1.6–3.9)	−1.47
		50 ≤ 69	125.3 (61.0–201.6)	65.1 (35.9–101.1)	−2.26	89.9 (51.4–132.0)	33.6 (19.6–50.1)	−3.39
		≥70	189.3 (77.7–346.6)	147.5 (75.2–244.8)	−0.86	197.0 (94.5–329.4)	137.6 (70.4–234.2)	−1.24
	Stroke	15 ≤ 49	7.8 (4.1–11.6)	8.4 (5.3–12.0)	0.23	5.5 (3.4–7.5)	4.7 (3.1–6.6)	−0.56
		50 ≤ 69	92.1 (45.6–143.4)	46.5 (26.6–70.0)	−2.36	66.5 (39.2–94.7)	28.9 (17.5–41.3)	−2.88
		≥70	113.2 (46.7–204.4)	83.8 (41.0–138.5)	−1.04	119.7 (47.5–195.0)	65.4 (33.4–108.0)	−2.09
	T2DM	15 ≤ 49	3.1 (1.6–4.5)	12.8 (8.4–17.6)	4.88	2.2 (1.4–3.1)	7.7 (5.4–10.4)	4.28
		50 ≤ 69	60.8 (32.5–90.3)	179.5 (112.7–255.2)	3.73	76.7 (50.4–100.7)	136.0 (91.3–187.5)	1.97
		≥70	56.3 (23.4–98.3)	329.9 (179.4–515.6)	6.19	90.6 (46.2–141.9)	362.8 (210.8–552.8)	6.10
Mozambique	IHD	15 ≤ 49	0.6 (0.1–1.6)	2.5 (1.1–4.8)	4.83	0.6 (0.2–1.2)	1.4 (0.6–2.5)	3.04
		50 ≤ 69	10.4 (2.0–26.2)	40.8 (16.9–71.8)	4.70	7.1 (1.6–16.1)	20.6 (9.4–36.0)	3.69
		≥70	22.0 (2.9–63.9)	68.9 (20.7–141.7)	3.94	20.9 (3.8–54.1)	57.4 (20.6–114.4)	3.48
	Stroke	15 ≤ 49	2.3 (0.5–5.4)	7.9 (3.5–13.5)	4.33	2.3 (0.7–4.7)	2.5 (2.3–7.5)	2.26
		50 ≤ 69	31.4 (6.2–78.9)	105.7 (44.0–178.5)	4.19	24.6 (6.1–53.7)	59.8 (28.6–100.6)	3.06
		≥70	38.8 (5.4–104.5)	106.1 (34.1–211.5)	3.47	49.1 (8.5–125.3)	110.6 (4.5–212.2)	2.80
	T2DM	15 ≤ 49	0.7 (0.1–1.6)	2.8 (1.3–4.6)	4.80	0.9 (0.3–1.7)	1.6 (0.9–2.5)	2.20
		50 ≤ 69	13.3 (2.8–32.1)	56.3 (25.6–91.4)	4.99	11.6 (3.0–24.8)	30.1 (15.5–48.9)	2.39
		≥70	23.2 (3.3–63.8)	76.6 (24.6–149.8)	4.12	21.7 (4.0–54.1)	56.4 (22.3–106.3)	4.12
Namibia	IHD	15 ≤ 49	2.0 (0.83.6)	3.8 (1.7–6.7)	2.30	1.5 (0.7–2.4)	1.6 (0.8–2.8)	0.33
		50 ≤ 69	52.4 (24.1–88.4)	80.0 (43.9–126.2)	1.47	40.4 (–22.3–64.3)	37.3 (21.1–59.4)	−0.27
		≥70	81.0 (32.3–155.5)	144.9 (64.1–252.7)	2.00	123.5 (58.0–210.5)	181.2 (98.2–292.9)	1.32
	Stroke	15 ≤ 49	3.5 (1.5–6.2)	4.7 (2.3–8.0)	1.01	4.7 (2.5–7.0)	3.0 (1.6–5.0)	−1.57
		50 ≤ 69	80.1 (37.0–139.0)	93.5 (53.4–141.1)	0.54	100.4 (57.4–154.2)	65.0 (39.7–102.8)	−1.50
		≥70	91.1 (35.9–177.8)	133.0 (56.2–223.3)	1.31	174.5 (80.2–292.8)	183.0 (101.8–292.3)	0.16
	T2DM	15 ≤ 49	1.2 (0.5–2.0)	2.4 (1.2–3.8)	2.40	1.7 (1.0–2.5)	1.6 (0.9–2.7)	−0.07
		50 ≤ 69	58.6 (28.7–94.9)	95.1 (59.2–143.7)	1.67	87.8 (54.0–126.6)	80.2 (50.7–119.0)	−0.31
		≥70	86.3 (33.7–167.1)	184.1 (83.3–320.6)	2.61	182.0 (87.1–301.5)	270.9 (163.1–405.8)	1.37
Seychelles	IHD	15 ≤ 49	6.8 (3.1–11.2)	10.9 (6.5–16.1)	–	3.2 (2.0–4.7)	3.3 (2.1–4.7)	–
		50 ≤ 69	75.9 (32.6–131.1)	72.6 (42.3–111.2)	–	47.4 (28.4–69.0)	33.2 (20.2–47.5)	–
		≥70	93.0 (23.9–208.2)	103.3 (41.3–184.5)	–	79.4 (26.8–165.0)	98.9 (39.4–181.9)	–
	Stroke	15 ≤ 49	8.1 (4.0–12.6)	10.8 (7.1–14.8)	–	5.3 (3.8–7.0)	4.3 (3.0–5.8)	–
		50 ≤ 69	70.3 (31.5–118.6)	56.3 (33.0–82.2)	–	48.5 (31.5–68.1)	27.3 (17.3–38.8)	–
		≥70	62.3 (16.2–135.8)	56.6 (23.4–102.2)	–	48.5 (17.3–95.0)	49.6 (21.2–90.0)	–
	T2DM	15 ≤ 49	0.6 (0.3–0.9)	2.7 (1.9–3.5)	–	0.7 (0.5–1.0)	2.2 (1.6–2.9)	–
		50 ≤ 69	13.2 (6.2–21.3)	28.9. (19.0–40.2)	–	20.7 (13.8–28.7)	29.7 (20.8–39.3)	–
		≥70	15.0 (4.0–31.5)	45.2 (18.6–77.2)	–	25.7 (9.2–48.8)	66.2 (29.5–112.2)	–
SA	IHD	15 ≤ 49	5.1 (3.0–7.8)	4.8 (3.1–6.6)	−0.22	4.1 (2.8–5.5)	2.3 (1.6–3.0)	−2.02
		50 ≤ 69	55.5 (33.0–80.8)	66.4 (42.8–92.6)	0.62	42.0 (28.2–54.9)	41.3 (29.2–53.6)	−0.06
		≥70	82.3 (33.5–142.5	151.3 (85.8–229.1)	2.10	138.0 (75.6–212.7)	186.3 (109.7–270.5)	1.04
	Stroke	15 ≤ 49	(6.9)4.3–9.7	5.1 (3.7–6.6)	−1.04	8.8 (6.6–11.0)	3.5 (2.7–4.5)	−3.13
		50 ≤ 69	(55.7)33.3–81.0	62.8 (43.383.9)	0.41	68.0 (47.5–86.9)	56.6 (41.7–69.8)	−0.63
		≥70	(60.3)27.6–107.7	104.7 (59.3–157.5)	1.90	123.6 (67.6–190.5)	159.2 (94.0–234.0)	0.87
	T2DM	15 ≤ 49	(2.8)2.0–3.6	3.8 (3.0–4.6)	1.02	3.6 (2.9–4.2)	2.9 (2.3–3.6)	−0.72
		50 ≤ 69	(56.1)38.1–75.3	108.5 (83.0–135.2)	2.27	82.0 (63.6–98.9)	113.2 (91.0–133.6)	1.11
		≥70	(86.5)43.0–139.1	253.6 (162.6–354.0)	3.71	186.6 (119.7–265.4)	368.4 (248.8–486.5)	2.35
ESwatini	IHD	15 ≤ 49	3.4 (1.6–5.8)	7.6 (3.7–13.3)	2.76	2.0 (1.1–3.2)	2.6 (1.2–5.0)	0.86
		50 ≤ 69	81.0 (42.2–128.4)	130.2 (75.7–198.8)	1.64	52.5 (32.1–77.0)	62.1 (32.3–104.3)	0.58
		≥70	139.9 (63.3–249.9)	227.2 (123.8–366.3)	1.67	219.2 (127.8–332.1)	261.0 (147.1–414.4)	0.60
	Stroke	15 ≤ 49	5.2 (2.7–8.3)	8.2 (4.1–13.5)	1.59	5.5– (3.3–7.9)	4.4. (2.1–8.1)	−0.72
		50 ≤ 69	119.2 (65.6–182.1)	151.3 (93.1–223.8)	0.82	119.8 (75.3–176.0)	95.0 (52.1–154.4)	−0.80
		≥70	164.5 (72.1–292.0)	234.3 (125.6–381.9)	1.22	285.2 (163.9–424.9)	281.5 (154.7–454.9)	−0.04
	T2DM	15 ≤ 49	3.0 (1.7–4.8)	10.0 (5.1–15.6)	4.09	2.8 (1.7–3.9)	4.2 (1.9–7.3)	1.45
		50 ≤ 69	129.8 (76.7–191.3)	315.6 (197.7–439.3)	3.06	138.5 (90.8–191.1)	194.5 (108.4–306.6)	1.17
		≥70	237.5 (111.5–399.8)	654.0 (370.1–966.0)	3.49	384.9 (239.4–543.1)	636.7 (365.0–940.6)	1.74
Tanzania	IHD	15 ≤ 49	1.3 (0.4–2.4)	2.3 (1.0–4.4)	2.09	0.5 (0.2–1.0)	1.4 (0.8–2.2)	3.22
		50 ≤ 69	12.7 (7.7–42.8)	37.3 (17.1–63.6)	1.71	10.7 (5.0–18.7)	22.3 (12.4–35.0)	2.53
		≥70	57.0 (17.3–122.2)	115.5 (48.0–214.0)	2.44	51.2 (18.1–100.7)	89.1 (37.8–158.1)	1.91
	Stroke	15 ≤ 49	3.7 (1.4–6.8)	4.2 (1.9–7.4)	0.36	3.7 (1.7–6.2)	4.7 (3.0–6.9)	0.78
		50 ≤ 69	44.4 (14.7–85.1)	46.6 (20.7–82.9)	0.17	57.0 (28.5–93.9)	70.8 (40.6–104.9)	0.75
		≥70	66.5 (19.3–139.6)	96.6 (40.04–178.3)	1.29	86.1 (28.7–162.9)	124.5 (53.0–213.9)	1.27
	T2DM	15 ≤ 49	1.0 (0.4–1.8)	1.8 (0.9–2.9)	1.87	1.0 (0.5–1.7)	1.6 (1.0–2.8)	1.48
		50 ≤ 69	23.0 (8.7–41.0)	38.1 (20.8–59.4)	1.74	(28.0)15.2–42.6	39.9 (26.9–55.0)	1.22
		≥70	47.8 (15.1–98.1)	95.5 (43.9–162.3)	2.39	(61.2)23.9–111.2	93.4 (47.0–155.1)	1.46
Zambia	IHD	15 ≤ 49	2.0 (0.7–3.8)	3.4 (1.6–5.8)	1.76	1.6 (0.6–3.1)	1.9 (0.9–3.2)	0.49
		50 ≤ 69	28.8 (9.8–56.2)	40.6 (19.4–68.6)	1.19	21.3 (8.0–40.8)	31.2 (17.0–49.5)	1.32
		≥70	45.1 (11.6–104.2)	82.1 (32.1–152.0)	2.06	53.6 (15.6–114.3)	70.5 (30.5–124.1)	0.94
	Stroke	15 ≤ 49	3.5 (1.3–6.3)	7.5 (3.8–12.3)	2.63	4.3 (1.9–7.5)	4.9 (2.7–7.5)	0.40
		50 ≤ 69	46.1 (16.0–87.7)	97.9 (49.4–156.1)	2.60	49.6 (20.1–88.1)	85.0 (48.9–128.3)	1.85
		≥70	56.1 (12.6–134.1)	136.9 (53.7–248.0)	3.08	79.0 (22.7–165.5)	148.7 (65.4–264.2)	1.18
	T2DM	15 ≤ 49	1.2 (0.5–2.0)	2.7 (1.5–4.2)	2.87	1.6 (0.7–2.6)	1.7 (1.0–2.5)	0.29
		50 ≤ 69	28.7 (10.8–51.6)	53.0 (29.5–80.8)	2.11	28.9 (12.8–48.6)	41.4 (26.6–59.4)	1.24
		≥70	45.1 (11.4–100.7)	99.8 (41.1–175.4)	2.74	52.9 (16.1–104.7)	80.4 (38.6–136.7)	1.45
Zimbabwe	IHD	15 ≤ 49	1.1 (0.4–2.0)	2.3 (0.9–4.2)	2.59	1.0 (0.6–1.6)	2.6 (1.4–4.5)	3.31
		50 ≤ 69	32.8 (12.6–59.1)	52.9 (23.0–89.5)	1.65	54.7 (31.9–82.2)	96.6 (54.9–145.9)	1.93
		≥70	58.5 (17.1–119.5)	85.4 (30.9–163.6)	1.30	148.2 (72.8–249.9)	217.4 (107.0–372.8)	1.32
	Stroke	15 ≤ 49	1.4 (0.6–2.5)	2.6 (1.1–4.9)	2.07	2.0 (1,2–3.1)	4.0 (2.3–6.4)	2.39
		50 ≤ 69	28.9 (10.9–54.2)	38.5 (17.1–68.2)	1.00	50.6 (31.6–75.0)	64.0 (37.8–96.8)	0.81
		≥70	34.6 (10.0–71.7)	46.0 (15.3–90.7)	0.98	84.0 (39.1–143.6)	110.4 (52.9–192.0)	0.94
	T2DM	15 ≤ 49	0.6 (0.3–1.0)	1.6 (0.7–2.7)	3.32	0.8 (0.5–1.1)	1.8 (1.1–2.7)	2.89
		50 ≤ 69	23.3 (9.5–38.6)	48.8 (23.7–77.8)	2.55	60.3 (40.5–83.7)	110.7 (69.4–157.3)	2.10
		≥70	29.4 (8.4–59.7)	58.9 (21.6–111.6)	2.40	109.5 (55.6–171.4)	200.0 (105.0–323.2)	2.08

## 4 DISCUSSION

A descriptive, epidemiological analysis of the prevalence of high BMI (overweight and obesity), stroke, IHD, and T2DM in 1990 and 2019 for the 16 SADC countries was conducted. Between 1990 and 2019 BMI increased by 0.7 in adult males, and by 1.0 in adult females. Obesity increased >1.5‐fold in adult females and nearly doubled in adult males, corresponding to an AROC of 0.64% and 1.15%, respectively. Obesity more than doubled in boys and girls. Overall, deaths due to IHD, stroke, and T2DM attributable to high BMI increased between 2.5 and 3.5‐fold. Countries with greatest increase in mean BMI in females tended to be the same countries with the greatest increases in mean BMI in males.

A previous GBD study reported an increase in prevalence of obesity globally, more in adults than in children and greater in women than in men.^24^ As found in the present study, the NCD Collaboration,^34^ BMI increased in all regions and age groups, and was higher, for women than men for the period 1975–2016.^35^ A similar increasing trend in the burden of obesity was observed in African countries over three decades earlier (1980–2014). Additionally, there was a strong positive linear correlation between mean BMI and prevalence of T2DM.^35^ Our study is the first to provide estimates of changes in overweight and obesity prevalence, deaths, YLLs, YLDs, and DALYs associated with high BMI, thereby providing a systematic understanding of the distribution of disease and time trends across SADC member countries. A better understanding of the past and current trends in the prevalence of high BMI burden would serve as guide for policymakers and other stakeholders in public health planning and resource allocation to enable the region to work toward achieving the SDG agenda by 2030.^21^

A major strength of our study is that it is based on all available data sources that could be accessed to estimate age‐standardized change in the disease burden over a period of 29 years; GBD studies use covariates and other analytic techniques that yield unbiased estimates whenever primary data are scarce for a particular variable. Age‐standardization facilitate inter‐country comparisons in instances where the population pyramids differ. Another major strength is all GBD estimates used by the research team adhere to the 14 GATHER which recommends making available statistical code, details on why some sources are used and others are not, and how primary data are adjusted.^23^ Full details of data sources, availability of data, and methods of dietary risk factors and BMI are described in detail elsewhere.^24^ Other strengths include the use of standardized GBD methodology for comparison across countries and years and robust analyses.

Our study revealed that between 1990 and 2019, age‐standardized mortality due to high BMI in males increased by 22.7% and 14.2% in females. A recent meta‐analysis, to the contrary, revealed reduced risk of mortality associated with the overweight BMI class compared with the normal BMI class and for class 1 or 2 obesity. Among studies in the meta‐analysis examining BMI change, increases in BMI demonstrated lower mortality risks compared with decreases in BMI. The apparent contradiction is that, unlike the present study, estimates included in the meta‐analyses were not standardized for age. Overweight BMI classification or a higher BMI value may be protective for all‐cause mortality, relative to normal BMI, in older adults. These findings demonstrate the potential need for age‐specific BMI cut‐points in older adults and further studies.^36^

### 4.1 Study limitations

Our study is not without limitations. General limitations of the GBD methodology are described elsewhere.^13^, ^14^, ^24^ In this particular case, limitations include incomplete medically certified cause of death systems in SADC countries and the non‐availability of traditional individual, social, and environmental risk factors for T2DM, IHD, and stroke such as health care access, urbanization, employment, physical inactivity, dietary risks, alcohol and cigarette use, and lipids, among others. These factors would make estimates presented in this paper more robust.

### 4.2 Recommendations

Individual and population‐wide interventions need to be established around strategies with a strength in disease management. Individual interventions must consist of prevention and treatment.^9^, ^37^, ^38^ For individuals with overweight or obesity, lifestyle modification targeting changes in diet and physical activity through a multidisciplinary approach could be the cornerstone of interventions for weight management.^38^ Health researchers and policymakers should develop targeted interventions to reduce consumption of certain foods classified as “harmful” and encourage people to eat health‐promoting foods. Where applicable, referral of patients for further interventions such as behavioral therapy and treatment—including pharmacotherapy and bariatric surgery—is recommended.^9^, ^37^, ^38^ Prevention is the ideal option in the SADC at present. Population approaches for overweight/obesity recommended for children should include restricting advertisements that promote consumption of unhealthy foods, providing healthy school meals, and encourage school‐based good nutrition education.^9^, ^37^

Other strategies should include use of the WHO STEPwise approach to NCD risk factor surveillance (STEPS) tool.^34^ The tool is used to collect data and measure NCD risk factors within the WHO STEPwise approach to surveillance. The STEPS Instrument covers three different levels or “steps” of risk factor assessment: Step 1 (questionnaire), Step 2 (physical measurements), and Step 3 (biochemical measurements).

## 5 CONCLUSION

The burdens of high BMI (overweight and obesity), stroke, IHD, and T2DM increased dramatically by 2019 compared to 1990. The prevalence of obesity more than doubled in adults and increased 1.7‐fold in children. The 2019 mean BMI for adult females in 7 of the 16 countries was greater than the overweight cut‐point of >25 kg/m^2^. The countries with greatest increase in mean BMI in females tended to be the same countries with the greatest increases in mean BMI in males. SADC countries do have a “window “of opportunity to halt and reverse these negative health outcomes before they become endemic and out of control.

## CONFLICT OF INTEREST

All authors report no conflict of interest
